# Sedimentary Nitrogen and Sulfur Reduction Functional-Couplings Interplay With the Microbial Community of Anthropogenic Shrimp Culture Pond Ecosystem

**DOI:** 10.3389/fmicb.2022.830777

**Published:** 2022-03-04

**Authors:** Renjun Zhou, Dongwei Hou, Shenzheng Zeng, Dongdong Wei, Lingfei Yu, Shicheng Bao, Shaoping Weng, Jianguo He, Zhijian Huang

**Affiliations:** ^1^State Key Laboratory of Biocontrol, Southern Marine Sciences and Engineering Guangdong Laboratory (Zhuhai), School of Marine Sciences, Sun Yat-sen University, Guangzhou, China; ^2^School of Life Sciences, Sun Yat-sen University, Guangzhou, China

**Keywords:** nitrogen cycle, sulfur cycle, functional coupling, GeoChip, sediment microbial community

## Abstract

Sediment nitrogen and sulfur cycles are essential biogeochemical processes that regulate the microbial communities of environmental ecosystems, which have closely linked to environment ecological health. However, their functional couplings in anthropogenic aquaculture sedimentary ecosystems remain poorly understood. Here, we explored the sediment functional genes in shrimp culture pond ecosystems (SCPEs) at different culture stages using the GeoChip gene array approach with 16S amplicon sequencing. Dissimilarity analysis showed that the compositions of both functional genes and bacterial communities differed at different phases of shrimp culture with the appearance of temporal distance decay (*p* < 0.05). During shrimp culture, the abundances of nitrite and sulfite reduction functional genes decreased (*p* < 0.05), while those of nitrate and sulfate reduction genes were enriched (*p* < 0.05) in sediments, implying the enrichment of nitrites and sulfites from microbial metabolism. Meanwhile, nitrogen and sulfur reduction genes were found to be linked with carbon degradation and phosphorous metabolism (*p* < 0.05). The influence pathways of nutrients were demonstrated by structural equation modeling through environmental factors and the bacterial community on the nitrogen and sulfur reduction functions, indicating that the bacterial community response to environmental factors was facilitated by nutrients, and led to the shifts of functional genes (*p* < 0.05). These results indicate that sediment nitrogen and sulfur reduction functions in SCPEs were coupled, which are interconnected with the SCPEs bacterial community. Our findings will be helpful for understanding biogeochemical cycles in anthropogenic aquaculture ecosystems and promoting sustainable management of sediment environments through the framework of an ecological perspective.

## Introduction

Biogeochemical cycles play irreplaceable roles in global ecosystem sustainability. The cycles are also strongly linked to environmental pollution (Nriagu and Coker, 1983; Kondratyev et al., 2004; Kuypers et al., 2018). For example, nitrogen and sulfur cycles are important biogeochemical processes in global ecosystems (Canfield and Farquhar, 2012; Kuypers et al., 2018). As environmental pollutants, nitrite and sulfite are formed from nitrogen dioxide and sulfur dioxide, respectively, compounds that are toxic to animal health (Akanji et al., 1993; Xu et al., 2021). Nitrite can be generated by the metabolism of amino acids from microbes (Neis et al., 2015), and sulfite is generated by the metabolism of methionine and cysteine, and from sulfates in response to bacterial lipopolysaccharides (Kabil et al., 2014). Both sediment nitrogen and sulfur compounds can be the causes of acidification of the aquatic and soil environments (Nriagu and Coker, 1983; Doney et al., 2007; Kissel et al., 2020). At the cellular level, those processes are generally coupled with microbial metabolism such as degradation processes of organic matter by microbes (Kleindienst et al., 2014), while the relationships between nitrite and sulfite generation and the environmental microbial community need further investigation.

Biogeochemical function couplings and their interactions with the microbial community are important ecological topics (Gao et al., 2021; Ma et al., 2021). Researchers have demonstrated that the connections among species, as well as between species and their habitats, have been disrupted due to the current dramatic global environmental changes, with far-reaching consequences for the future trajectory and functioning of entire ecosystems (Tylianakis et al., 2008; Valiente-Banuet et al., 2015; Ochoa-Hueso et al., 2021). The exploration of biogeochemical couplings and their interactions with biotic and abiotic factors is needed to understand the regeneration of sustainable ecosystems (Wubs et al., 2016), as well as the future ecological intensification of agroecosystems (Toju et al., 2018).

Since the aggravation human activities, the topic of ecosystem health has received increasing attention (Zhu et al., 2019). It is critical to understand microbial identity, diversity, function, and the interactions between these factors and their relative impact on ecosystem functioning. From the perspectives of “One Health” and “Planet Health” (Zhu et al., 2020), concerns have arisen about shifts of soil ecosystems as a result of human activities. From the basic perspective of cellular metabolism to ecosystem structure and nutrient cycling, elements are biologically coupled through their effects on the biochemical reactions that regulate primary production, respiration, and decomposition in global ecosystems and these effects are connected to environmental health (Yuan and Chen, 2015). Among the affected ecosystems, sediment biogeochemical cycles are the foundation of ecosystem functions, as they affect nutrient and energy flows that regulate productivity within both terrestrial and aquatic ecosystems (Wardle et al., 2004). Diverse results regarding interactions between functional coupling and soil taxa have been observed. A low connectivity between soil functions and the microbial community has been reported from Mediterranean-type natural grassland soil (Gao et al., 2021), while a strong relationship between functions and microbes has been observed in forest soils (Ma et al., 2021). In aquatic environments, reports have profiled the weak influence of the taxonomic composition within marine ecosystems (Louca et al., 2016). Nevertheless, the ecological patterns of anthropogenic ecosystem functional couplings and their taxonomic interconnections are still unclear.

As one of the intensive agroecosystems, inland aquaculture ecosystems are influenced by human production activities that differ from natural ecosystems. According to the statistics recorded by the Fishery and Agriculture Organization (FAO), the reclamation of aquaculture ponds covered 1.27 million hectares, with the amount of aquaculture products being 31,304,026 tons on the Chinese mainland in 2017 (FAO, 2017). Although the expansion of inland aquaculture has contributed to global food security and the sustainability of wild fishery ecosystems, scientific management is still required for dealing with the potential challenges from farming (Naylor et al., 2021). For instance, the shrimp culture pond ecosystem (SCPE) is an important part of the inland aquaculture ecosystems that are continuously fed with additive nutrients (fodder) during farming, and those nutrients that are not completely consumed by the cultured animals will settle into the sediments. The accumulated redundant nutrients affect the microbial community structure (Carrero-Colón et al., 2006; Mello et al., 2016; Hou et al., 2018), and thus potentially alter the ecological functions of the sediment environment. These ecological shifts may challenge the health of both the environment and aquaculture species. For the environment, the shrimp aquaculture sediment promotes the production of bioavailable ammonium by dissimilatory nitrate reduction that can aggravate nitrogen loading in coastal wetlands (Gao et al., 2019). For aquaculture species, shrimps are sensitive to the environment (Huang et al., 2020), and the enrichment of hazardous substances, such as nitrite and sulfite, is thought to be the cause of shrimp diseases (Bonerba et al., 2013; Hou et al., 2017; Valencia-Castañeda et al., 2018). Previous studies reported that the microbial community composition in SCPE sediments changed dramatically during shrimp culture (Zhou et al., 2021), while the ecological functional dynamics and their relationship with the microbial drivers in shrimp culture sediment remain unknown.

Herein, we report the results of an investigation of the functional genes and microbial community in sediments at different stages of shrimp culture. SCPE sediments are under long-term feed deposition during shrimp culture activities. Thus, abundant nutrients could be the dominant factor governing the sediment ecosystem. In this study, we hypothesized that (H1) sediment functional genes varied along with the shrimp culture at different culture stages and that (H2) sediment functional couplings connected with the microbial community. To verify these hypotheses, we conducted functional gene detection using a micro-array approach, with microbial community exploration *via* 16S rRNA amplicon sequencing. The results of this study will improve our understanding of sediment functional gene dynamic patterns during aquaculture activities and will be helpful for the management of sediment environments.

## Materials and Methods

### Sampling and Measurement of Physicochemical Parameters

From June to August 2017, sediment samples were collected from four commercial shrimp (*Penaeus vannamei*) culture ponds in Zhuhai (113.22°E, 22.37°N), Guangdong, China. The ponds were approximately uniform in size (2,000 m^2^) with about 0.7 m of water depth. Shrimp larvae were introduced into the ponds. The length of shrimp averaged 0.8 cm and the stocking density was 100,000 shrimp for each pond in zero-water exchange culture with major protein-source feed every day. Sediment samples from surface sediment were collected in triplicate using a core sampler (Wei et al., 2021), from the 2nd day after the shrimp larvae were introduced into the pond to the 58th day of culture, with an interval of approximately 8 days (designated as Day 02, Day 13, Day 21, Day 31, Day 42, Day 51, and Day 58). Sediments from each pond were pooled and placed into sealed sterile plastic bags in a cooler with ice for transport to the laboratory. Finally, a total of 27 samples were collected. The samples were homogenized and dried with a freeze dryer (LGS-50F, Beijing, China). The other sediments were stored at −80°C for DNA extraction.

The sediment pH value was measured by a pH meter (ZD-06, ZD instrument, China). Oxidation–reduction potential (ORP) was measured by an ORP meter (Sanxin-5,041, Shanghai San-Xin Instrumentation Inc., China). Total phosphate (TP) and total nitrogen (TN) were measured as described previously (Hou et al., 2021; Huang et al., 2021). Sediment ammonium nitrogen (NH_4_^+^-N), nitrite nitrogen (NO_2_^−^-N), and nitrate nitrogen (NO_3_^−^-N) were measured by spectrophotometric methods using an auto discrete analyzer (Model CleverChem 380, DeChem-Tech, Germany). Sediment total carbon (TC) and total organic carbon (TOC) were analyzed by a TOC analyzer (Aurora 1,030 W, OI, United States). The sediment physicochemical information at different shrimp culture stages is presented in Supplementary Table S1.

### DNA Extraction and 16S rRNA Amplicon Sequencing

Genomic DNA in sediment samples was extracted with a Power Soil DNA Isolation Kit (QIAGEN, Hilden, Germany) according to the manufacturer’s protocol. The PCR products were equally combined and paired-end sequenced (2 × 300) by an Illumina MiSeq platform (Illumina, San Diego, United States) from Majorbio Bio-Pharm Technology Co. Ltd. (Shanghai, China). The universal primer pair of 338F and 806R (5′-ACTCCTACGGGAGGCAGCAG-3′ and 5′-GGACTACHVGGGTWTCTAAT-3′) was used to amplify the V3-V4 regions of the bacterial 16S rRNA gene.

Sequencing data from this study have been submitted to the NCBI BioProject under accession number PRJNA686005.

### Bioinformatic Analysis

The 16S rRNA amplicon paired-end sequences were merged using FLASH (Magoc and Salzberg, 2011), and the merged sequences were processed with the Usearch pipeline (version 11.0.667). In brief, the sequences with ambiguous bases were discarded if the expected error value was higher than 1.0 (Edgar, 2010). Chimeric sequences were removed using the UCHIME algorithm (Edgar et al., 2011). Sequences with a distance-based identity of 97% or greater were grouped into operational taxonomic units (OTUs) using the UPARSE-OTU algorithm (Edgar, 2013). The most abundant sequence from each OTU was selected as representative and then was taxonomically assigned against the Silva SSU database 132 using the RDP Classifier algorithm (http://rdp.cme.msu.edu/) that enables each identified OTU to have a close relative.

### Detection and Analysis by GeoChip

We used a comprehensive functional gene array (GeoChip 5.0) to analyze all samples, comprising a total of 57,603 gene variants from 373 functional gene families involved in C, N, S, and P cycling and six other functional categories, as well as 44 classified functional subcategories.^1^ The detection of functional genes in shrimp culture pond sediment was performed by MAGIGENE (Guangzhou, Guangdong, China). The raw data were first normalized by Log10 conversion and then scaled by the average. These data represent relative abundances based on signal intensities that were normalized to the total intensities determined for the arrays for each sample (Morrison et al., 2016), and further analysis of sediment functional gene dynamics at different shrimp culture stages was conducted based on variation of gene relative abundances (He et al., 2020).

### Structural Equation Modeling

We examined the relationships among sediment physicochemical properties, the bacterial community, and nitrogen and sulfur reduction functions using a structural equation modeling (SEM) framework. Within the model, physicochemical properties were linked by correlations. The nutrient addition effects and their effects on the bacterial community function profiles were modeled with causal relations (directed paths). The SEM framework was fitted using the R sem function from the Lavaan package (version 0.6–8; Rosseel, 2012). The profiles of the microbial community and functional genes were calculated using eigenvalues derived from Bray-Curtis distance-based non-metric multidimensional scaling (NMDS) calculations using one dimension. To avoid any model-fit deviation due to scale differences between variables, all explanatory variables were centered and divided by two standard deviations for our analyses using the R rescale function from the arm package (version 1.11–2). The model fit to the data and model quality were assessed using three complementary indices: (1) the root mean square error of approximation (RMSEA), (2) the comparative fit index (CFI), and (3) the standardized root mean squared residual (SRMR). Model fits were considered acceptable when the RMSEA was <0.06, the goodness-of-fit index (GFI) was >0.90, the CFI was >0.9, and the SRMR was <0.08 (You et al., 2014).

### Statistical Analysis

The species richness, Shannon and inverse Simpson indices were calculated using the picante package (version 1.8.2) in R (Kembel et al., 2010). The significance (value of *p*) and explained sum of squares (R-squared value) of the fitted curves from linear regression were summarized using R version 3.6.1 (Revolution Analytics, United States; Zhou et al., 2020). The expected increment or decrement of the gene dynamics during shrimp culture was represented by the fitted curve slope, and the values of change were calculated according to the estimated value from the final shrimp culture stage (Day 58). Bray–Curtis similarity calculations and NMDS of each sample were conducted using vegan (version 2.5–6) in R (Anderson, 2001). The Mantel test was conducted to analyze the relationships among functional genes, microbial communities, and environmental factors (Anderson, 2001). Distance-based redundancy analysis (db-RDA) and variation partitioning analysis (VPA) were used to examine the explanatory power of microbial communities, environmental factors, and accumulated feed nutrients in relation to functional genes (Yergeau et al., 2010; Zhu et al., 2017). The normalized stochasticity ratio of the microbial community was calculated using the NST package (version 3.0.3) based on the weighted Bray-Cutis distance (Ning et al., 2019). All the values of *p* generated from multiple correlation analysis were adjusted by the false discovery rate (FDR) algorithm (Benjamini and Hochberg, 1995). The data were normalized into Z-score standardized values using scale function in R.

## Results

### Functional Gene Composition and Diversity in SCPE Sediments

**Figure 1:**
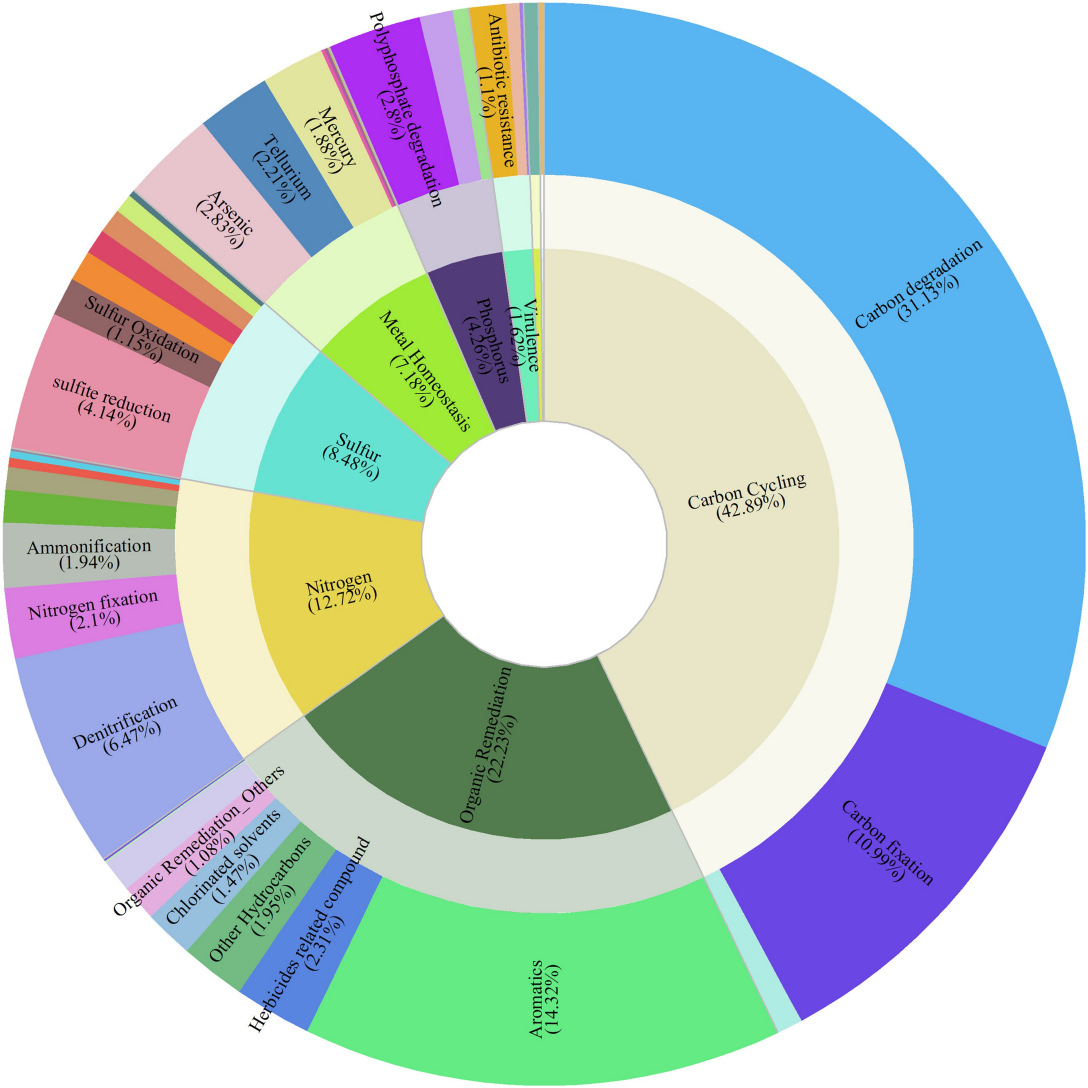
Composition of functional gene categories and gene subcategories. The inner circle represents the gene category; the outer circle represents the gene subcategory.

A total of 32,688 detected gene variants, involved in eight classified gene categories, 44 subcategories, and covering 358 functional gene families, were derived by GeoChip 5.0 (Table 1). The category of carbon cycling was the dominant function, accounting for 42.89% of the functional gene abundance. Organic remediation accounted for 22.21%, followed by nitrogen (12.72%), sulfur (8.48%), metal homeostasis (7.18%), and phosphorus (4.26%) related genes. The main function in the subcategory was carbon degradation that accounted for 31.13% of the functional gene abundance. Aromatics-related genes accounted for 14.32%, followed by carbon fixation (10.99%), denitrification (6.47%), sulfite reduction (4.14%), and arsenic (2.83%) and polyphosphate degradation (2.8%) subcategory functions (Figure 1). Bacteria contributed 88.01% of the microbial functional genes, and eukaryotes accounted for 8.55% (Supplementary Figure S2). Proteobacteria accounted for 40.36%, followed by Actinobacteria (17.33%). The sediment microbial functional diversity was also assessed (Supplementary Figure S2). The functional gene richness of temporal shrimp culture pond sediments ranged from 26,979 to 30,704, and their Shannon indices ranged from 10.19 to 10.32. Their inverse Simpson indices ranged from 26,223.52 to 29,684.06.

**Table 1:** The richness of functional gene categories detected at different culture stages.

Gene category (richness)	Day 02	Day 13	Day 21	Day 31	Day 42	Day 51	Day 58	Total
Carbon cycling	12,473	13,163	13,402	13,501	12,261	12,453	12,562	14,192
Organic remediation	6,357	6,615	6,710	6,742	6,216	6,352	6,380	7,017
Nitrogen	3,674	3,894	3,968	3,987	3,601	3,700	3,717	4,208
Sulfur	2,474	2,617	2,662	2,706	2,417	2,482	2,494	2,832
Metal homeostasis	2063	2,176	2,202	2,212	2040	2082	2,100	2,320
Phosphorus	1,231	1,288	1,312	1,316	1,197	1,221	1,239	1,378
Virulence	464	492	489	499	460	465	467	518
Secondary metabolism	49	52	50	52	50	48	49	54
Other	133	148	150	156	133	134	134	169
Total	28,918	30,445	30,945	31,171	28,375	28,937	29,142	32,688

### Abundance of Element Metabolism Functions in SCPE Sediments

The relevant nutrient metabolism functions were assessed, including the functions of carbon, nitrogen, phosphorus, and sulfur. For phosphorus metabolism, the signal intensity of the polyphosphate degradation gene *ppx* ranged from 758.20 to 870.08. The intensity of the polyphosphate synthesis gene *ppk* ranged from 261.84 to 310.20, and the values for the phytic acid hydrolysis gene phytase ranged from 126.78 to 147.94 (Supplementary Figure S3A). The carbon metabolism functional genes were classified into three subcategories, methane metabolism, carbon fixation, and carbon degradation functions. Methane metabolism genes, such as *mcr*A (113.39–131.99) and *pmo*A (66.92–88.52) as well as the methanogenesis gene *mmo*X (22.45–28.95), were identified. For the carbon fixation function, Calvin cycle genes were the most abundant, ranging from 1875.81 to 2129.89, followed by bacterial microcompartment-related function (451.74–520.00), reductive acetyl-CoA pathway-related genes (420.71–491.82), and reductive tricarboxylic acid cycle genes (102.63–125.35). The carbon degradation-related functional genes were sorted by the difficulty of degrading target compounds (Supplementary Figure S3B). The most abundant carbon degradation gene was the alpha-amylase gene *amy*A, which ranged from 2534.73 to 2742.79, followed by the chitinase gene (626.68–731.54), acetylglucosaminidase (396.11–458.61), and the alpha-L-arabinofuranosidase gene *ara* (371.62–424.66).

As for nitrogen metabolism-related genes, the genes related to denitrification had the highest signal intensity; these included *nar*G (661.56–734.93), *nos*Z (411.80–481.86), *nir*S (315.84–373.53), and *nir*K (219.19–332.20), followed by the nitrogen fixation gene *nir*H (558.15–665.77, Supplementary Figure S3C). The sulfite reduction-related genes had the highest signal intensity; these included *dsr*A (559.65–665.41), *dsr*B (408.92–481.06), and *sir* (131.69–154.46), followed by the reduction gene *cys*J (252.83–290.43, Supplementary Figure S3D).

### Gene Dynamics of Nitrogen and Sulfur Metabolism in Sediments During Shrimp Culture

**Figure 2:**
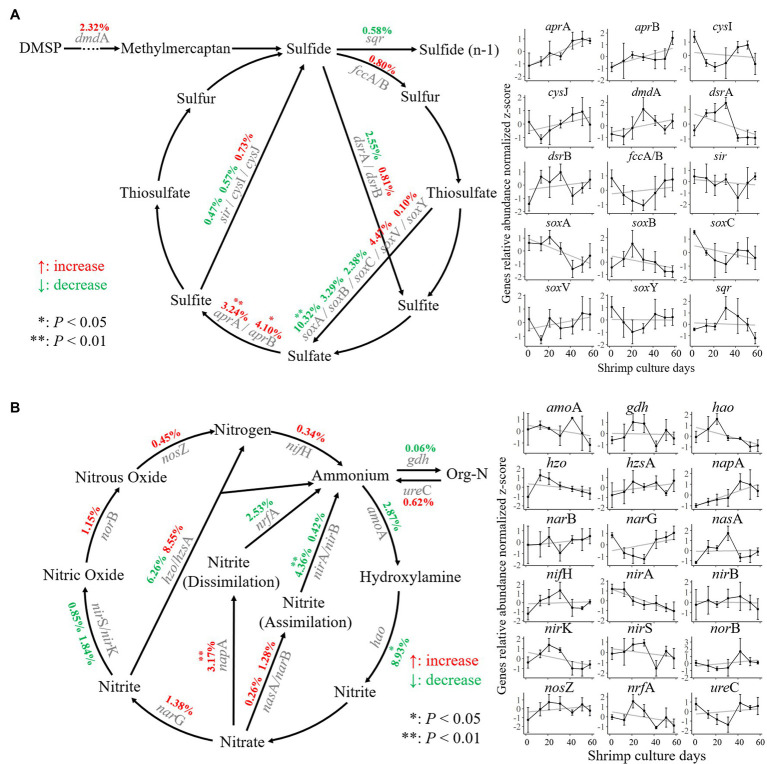
Nitrogen and sulfur cycling in the shrimp pond sediments. **(A)** Changes in the abundance of sulfur cycling-related genes. **(B)** Changes in the abundance of nitrogen cycling-related genes at different stages of culture. The central points and deviation bars indicate the mean ± SD.

The variation in functional genes related to nitrogen and sulfur metabolism in sediments was assessed during shrimp culture activity (Figure 2). There were 2,832 sulfur metabolism genes belonging to 15 gene families were identified that could be classified into seven reaction processes according to the sulfur cycle (Figure 2A). Within these aspects of metabolism, the abundance of sulfate reduction genes *apr*A and *apr*B was significantly increased (*p* < 0.05) by 3.24 and 4.10%, respectively. During the shrimp culture, the abundance of the sulfur-oxidizing protein gene *sox*A controlling oxidation of thiosulfate into sulfate decreased significantly by 10.32% (*p* < 0.05).

A total of 4,208 nitrogen metabolism-related genes covering 18 gene families were identified. Those could be classified into 14 reaction processes according to the nitrogen cycling model (Figure 2B). Within these processes, the abundance of the nitrate reductase gene *nap*A was significantly increased (*p* < 0.05) by 3.17% during the shrimp culture, while the abundance of the hydroxylamine dehydrogenase gene *hao* and the ferredoxin-nitrite reductase gene *nir*A was decreased significantly (*p* < 0.05) by 8.93 and 4.36%, respectively.

### Taxonomic Responses to Nitrate and Sulfate Reduction in Sediments During Shrimp Culture

**Figure 3:**
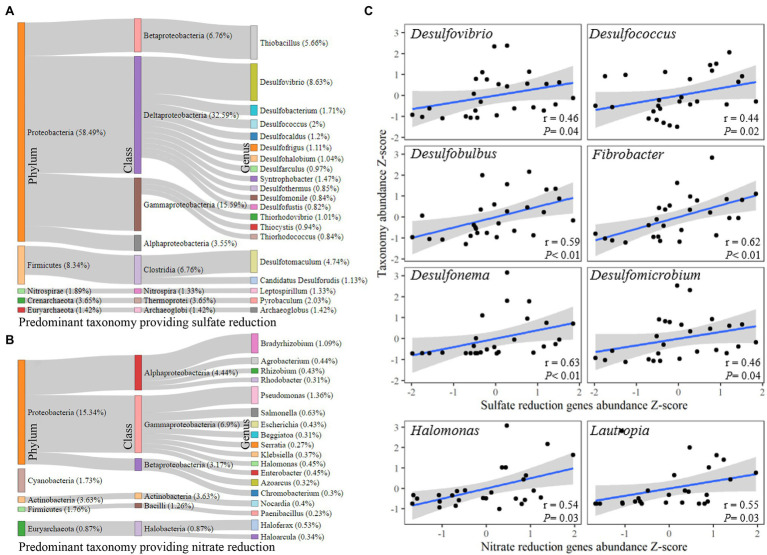
Taxonomic response to nitrate and sulfate reduction. **(A)** Taxonomic relationships with sulfate reduction. **(B)** Taxonomic relationships with nitrate reduction based on functional gene host (source). **(C)** Bacterial genera correlated with sulfate and nitrate reduction according to 16S amplicon operational taxonomic units (OTUs). The correlation analysis was conducted using Spearman’s rank correlation.

In order to identify the taxonomic responses responsible for the results regarding the significant shifts in sediment nitrogen and sulfur reduction gene abundances, the corresponding taxa derived from amplicons was assessed (Figure 3). There were 67 genera in 11 phyla found to be the carriers of sulfate reduction-related genes, and the Proteobacteria (58.49%) phylum made the largest contribution, followed by Firmicutes (8.34%). *Desulfovibrio* (8.63%) was the most common genus carrying sulfate reduction genes, followed by *Thiobacillus* (5.66%) and *Desulfotomaculum* (4.74%; Figure 3A). There were 111 genera in eight phyla that carried nitrate reduction-related genes, and the Proteobacteria (15.34%) phylum also took the most contribution, followed by Actinobacteria (3.63%). *Pseudomonas* (1.36% of the classified genus) was the leading contributor in carrying sulfate reduction-related genes, followed by *Bradyrhizobium* (1.36%; Figure 3B). After assessing the corresponding genera, *Desulfovibrio*, *Desulfococcus*, *Desulfobulbus*, *Fibrobacter*, *Desulfonema*, and *Desulfomicrobium* were found to be significantly correlated with the abundance of sulfate reduction genes (*p* < 0.05), and *Halomonas* and *Lautropia* had significant correlations with the abundance of nitrate reduction genes (*p* < 0.05; Figure 3C).

### The Structural Dynamics of Sediment Functional Genes and the Microbial Communities During Shrimp Culture

**Figure 4:**
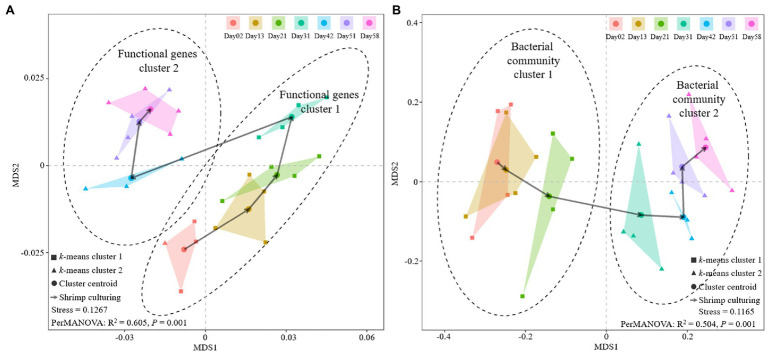
Variation of sediment functional gene structure and the bacterial communities at different shrimp culture stages. Non-metric multidimensional scaling (NMDS) analyses of **(A)** functional gene structure and **(B)** bacterial communities at different shrimp culture stages.

A Bray-Curtis distance-based NMDS analysis was conducted on the sediment microbial community and functional gene structure. The results showed a clear path across the centroid of each shrimp culture period, both in microbial community and functional genes (Figure 4). Moreover, the results of *k*-means algorithm analysis indicated that both microbial community and functional gene structure could be clustered into two significantly different culture periods (PerMANOVA, *p* = 0.001). Nevertheless, functional genes structure at Day 31 was clustered into the first period (Figure 4A), while the microbial community structure in Day 31 was clustered into the second period (Figure 4B). The results of temporal distance decay suggested that both functional genes and 16S bacterial community structure varied significantly during the shrimp culture. The linear regression slopes implied the turnover rates of the genes and bacterial community within the culture periods, suggesting that the turnover rates of the bacterial community were over 10 times higher than those of functional genes (Supplementary Figure S4).

### Relationships of Sediment Functional Genes With the Microbial Community and Environmental Factors

The sedimentary physicochemical parameters of ORP (−291.25–-170.5 mV), TC (19.66–26.2 mg·g^−1^), TOC (16.43–24.44 mg·g^−1^), TP (0.49–1.32 mg·g^−1^), NO_2_^−^-N (0.8–6.3 mg·kg^−1^), and NH_4_^+^-N (27.23–69.37 mg·kg^−1^) showed increase, while pH (6.47–4.6) was decreased during the shrimp culture (Supplementary Table S1).

**Figure 5:**
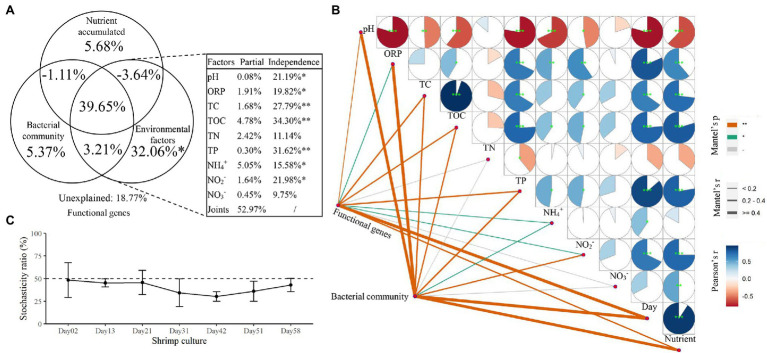
The relationships of functional genes with additive nutrients, environmental factors, and the bacterial community. **(A)** Variation partition analysis (VPA) shows the effects of environmental factors, additive nutrients, and the bacterial community on the functional genes. **(B)** Interactions among functional genes, the bacterial community, and environmental factors. **(C)** Stochasticity analysis of the bacterial community. *p < 0.05 and **p < 0.01.

The results of VPA indicated that microbial community structure could explain 46.12% of the variation in functional genes, and the values of nutrient accumulation and environmental factors were 40.58 and 71.28%, respectively. All factors together explained 81.23% of the variation in functional genes, with an 18.77% unexplained portion. For sediment environmental factors, pH, ORP, TC, TOC, TP, NH_4_^+^-N, and NO_2_^−^-N had significant explanatory power in relation to functional genes (*p* < 0.05, Figure 5A) and were also correlated both with functional genes and the microbial community according to the results of the Mantel test (Figure 5B). The results of microbial community stochasticity analysis indicated that the microbial community assembly in shrimp pond sediments was driven more strongly by deterministic processes than by stochasticity during the shrimp culture (Figure 5C). A close correlation has also been observed between the bacterial community and functional genes (Mantel test, Spearman’s *ρ* = 0.343, *p* = 0.002).

### Physicochemical Properties and Microbial Community Influence on Nitrogen and Sulfur Reduction Functions

**Figure 6:**
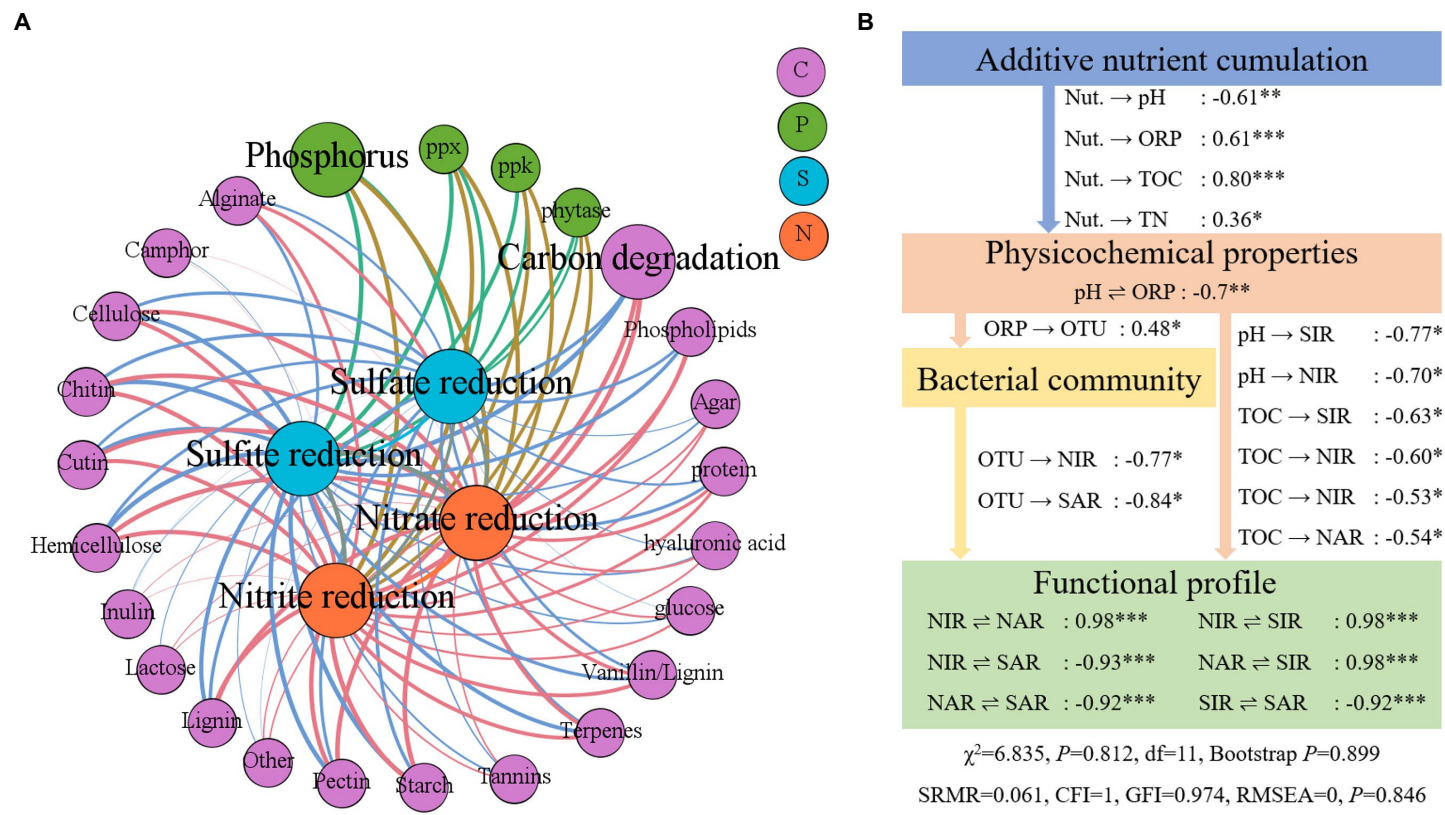
Influence of added nutrients, environmental factors, bacterial community, and metabolism other elements on nitrogen and sulfur reduction gene structure. **(A)** Mantel test for genes involved in carbon degradation, phosphorus metabolism, and nitrogen and sulfur reduction. **(B)** A structural equation model (SEM) depicts the influence paths from added nutrients, environmental factors, and the bacterial community. NIR, nitrite reduction; NAR, nitrate reduction; SIR, sulfite reduction; SAR, sulfate reduction. *p < 0.05, **p < 0.01, and *** *p* < 0.001.

A Mantel test-based network was constructed to evaluate the relationships of nitrogen and sulfur to carbon degradation and phosphorus metabolism using a threshold of Spearman’s coefficient *ρ* ≥ 0.5. A total of 29 nodes, with 104 links, including 18 classes of carbon degradation function and three classes of phosphorus function, were found to be correlated with nitrogen and sulfur reduction (Figure 6A). Among the nodes of the network model, carbon degradation was strongly correlated with nitrate reduction (Mantel test, Spearman’s *ρ* = 0.975, *p* = 0.001), followed by the connection between starch degradation and nitrate reduction (Mantel test, Spearman’s *ρ* = 0.972, *p* = 0.001). Meanwhile, the total carbon degradation and phosphorus metabolism functions were also correlated with sulfur and nitrogen reduction. According to the SEM analysis, a path of effect from nutrient accumulation to nitrate and nitrite reduction functional genes, *via* sediment physicochemical properties and the bacterial community, was presented (Figure 6B). The additive nutrients accumulated in the sediments had direct effects on the pH, ORP, TOC, and TN (SEM regression, *p* < 0.05). Sediment ORP had a direct effect on the bacterial community, and TOC had a direct effect on nitrogen and sulfur reduction (SEM regression *p* < 0.05). The bacterial community had a direct effect on sulfate and nitrite reduction genes (SEM regression *p* < 0.05). Meanwhile, the structures of nitrogen and sulfur reduction genes were highly correlated (SEM regression, *p* < 0.05).

## Discussion

Biogeochemical cycles are essential to the planet’s ecosystems, especially to sediment ecosystems (Aufdenkampe et al., 2011), while little is known about the patterns of biogeochemical functional coupling and their relationship with bacterial communities in anthropogenic sediments. This study found that sediment nitrogen and sulfur reduction functional genes; sediment functional genes were coupled, which were highly correlated with the microbial community.

### Variation of Functional Genes With the Microbial Community

The aquaculture ecosystem is a typical system that experiences frequent fluctuations in environmental variables caused by production activities such as nutrient supplementation and water regulation. Our results showed that most of the environmental variables were correlated with bacterial community and functional gene structures (Figure 5B, Mantel test, Spearman’s correlation, *p* < 0.05). Moreover, the shifts of bacterial community and functional gene structure were both discernable at different shrimp culture stages (Figure 2; PerMANOVA, *p* < 0.05), indicating that shrimp farming activities profoundly altered the bacterial component and microbial function of the SCPE sediments. The analysis of temporal distance decay suggested the turnover rates of the bacterial community structure were over 10 times higher than those of the functional gene structure (Supplementary Figure S3; linear regression *p* < 0.05), in despite of a trend of resilience observed in the functional turnover pattern. This finding suggested that functional genes were slower than the bacterial community in responding to the surrounding environment. The simultaneous but slower turnover of functional gene profiles compared with the bacterial community also corresponds to the concept of functional redundancy in microbial systems (Louca et al., 2018; Gao et al., 2021).

Nitrite and sulfite are environmental pollutants. They can be generated from nitrate and sulfate reduction by bacteria (Kaster et al., 2007; Zhou and Xing, 2021). Our study found that all the nitrate reduction genes (*nap*A, *nar*G, *nas*A, and *nar*B) increased along with abundance of sulfate reduction genes (*apr*A and *apr*B), whereas the abundances of most nitrite reduction genes (*nir*S, *nir*K, *hzo*, *nrf*A, *nir*A, and *nir*B) and sulfite reduction genes (*sir* and *cys*I) decreased during shrimp culture (Figure 3). The increase in the nitrate and sulfate reduction gene abundances implied the enhancement of reduction function. Meanwhile, a number of bacterial taxa were found to have significant responses to these shifts. *Halomonas* and *Lautropia* are facultatively anaerobic bacteria (Vreeland et al., 1980; Gerner-Smidt et al., 1994) that were highly correlated with the abundance of nitrate reduction genes. *Desulfovibrio*, *Desulfococcus*, *Desulfobulbus* (strictly anaerobic), *Fibrobacter*, *Desulfonema*, and *Desulfomicrobium* are also anaerobic bacteria (Gokarn et al., 1997; Warren et al., 2005; Kleindienst et al., 2014) that were correlated with the abundance of sulfate reduction genes. The significant increase in nitrate reduction gene abundance explains the increased concentration of nitrites in the sediments found in this study (Supplementary Table S1). These results may be due to the anaerobic environment in sediments that facilitate the reduction reactions of nitrates and sulfates (Baldwin and Mitchell, 2012), and the results are in agreement with a previous report concerning the nitrate reduction enhancement in shrimp pond sediments (Gao et al., 2019).

### Functional Genes Coupling and Relationship With the Microbial Community

In SCPEs, the additive nutrient supply for shrimp growth is the fundamental disturbance to the ecosystem. Carbon is the primary component of nutrients and its degradation metabolism drives other elements (Boroughs and DeBerardinis, 2015). Our study also showed a functional coupling between the metabolism of elements. The degradation of carbon and phosphorus was highly correlated with the nitrogen and sulfur reduction in functional gene structure, indicating the close functional coupling between microbial metabolism genes. The results implied that the additive nutrient degradation could be the essential source of nitrate and sulfate reduction.

The SCPE sediments show highly functional gene structural coupling and a close relationship with the microbial community, and the SEM analysis identified the influence paths from nutrients to specified microbial functions. The results of the SEM corresponded to those of the Mantel test (Figure 6). Nitrogen and sulfur reduction gene structures were highly correlated (*p* < 0.05, both SEM regression and Mantel test with Spearman’s rank correlation), thus emphasizing the coupling close coupling between them. In comparison, a grassland soil ecosystem showed bacterial-functional decoupling (Gao et al., 2021), with a weak interplay between community composition and functioning of lake and marine bacteria (Langenheder et al., 2005; Louca et al., 2016). Microbial functional genes and community structure are both sensitive to environmental factors (Gao et al., 2021), especially for environments under direct production activities; this could well explain the conspicuous coupling of microbial function and composition observed in this study. In comparison with natural and anthropogenic ecosystems, the differences in results regarding interactions between functional genes and microbial composition are most likely explained by the environments, specifically whether they receive additive nutrients or not. Meanwhile, the results of the microbial assembly process and SEM analyses demonstrate that aquaculture farming activities drive the changes in environmental factors, facilitate the microbial selective process within SCPE sediments, and result in shifts in the bacterial community and functional gene structure. In summary, the excessive nutrients consumed during the aquaculture activities might promote shifts in ecological function genes and lead to the enrichment of nitrite and sulfite pollutants in aquaculture sediments.

## Conclusion

This study investigated sediment functional genes in SCPEs using GeoChip gene array analysis. The results portrayed the relative comprehensive nutrient metabolism function in sediments, with signals of nitrate and sulfate reduction functional enhancement, and nitrite and sulfite reduction functional gene abundance decreased during shrimp culture. The SCPE sediment shows highly functional gene structural coupling, and ecosystem functions showed an interplay with microbial community composition. These results imply that the anthropogenic feeding process has profound impacts on the bacterial community and has led to changes in the ecological functions of the sediment environment. Our findings may be helpful for understanding biogeochemical cycles under human influence and for promoting sustainable management of the sediment environment through the framework of an ecological perspective.

## Data Availability Statement

The datasets presented in this study can be found in online repositories. The names of the repository/repositories and accession number(s) can be found in the article/Supplementary Material.

## Author Contributions

DH, SZ, DW, LY, and SB collected the samples and performed the experiments. RZ analyzed the data and wrote the manuscript. SW, JH, and ZH contributed to the conception of the work. ZH was primarily responsible for the final content. All authors contributed to the article and approved the submitted version.

## Funding

This work was financially supported by the China Agriculture Research System of MOF and MARA, the China-ASEAN Maritime Cooperation Fund, China-ASEAN Center for Joint Research and Promotion of Marine Aquaculture Technology, Key research and development projects in Guangdong Province (2020B0202010009 and 2021B0202040001), Guangdong MEPP Fund [No. GDOE (2019) A21], and the Southern Marine Science and Engineering Guangdong Laboratory (Zhuhai; SML2021SP203).

## Conflict of Interest

The authors declare that the research was conducted in the absence of any commercial or financial relationships that could be construed as a potential conflict of interest.

## Publisher’s Note

All claims expressed in this article are solely those of the authors and do not necessarily represent those of their affiliated organizations, or those of the publisher, the editors and the reviewers. Any product that may be evaluated in this article, or claim that may be made by its manufacturer, is not guaranteed or endorsed by the publisher.

## Supplementary Material

The Supplementary Material for this article can be found online at: https://www.frontiersin.org/articles/10.3389/fmicb.2022.830777/full#supplementary-material

Supplementary Table S1 Physicochemical information of shrimp culture pond ecosystem sediment environment at different shrimp culture stages.

Supplementary Figure S1 Taxonomic composition of functional gene host.

Supplementary Figure S2 Alpha-diversity of functional genes. The columns and deviation bars indicate the mean ± SD.

Supplementary Figure S3 The signal intensity of biogeochemical functional genes in GeoChip, **(A)** phosphorous, methane metabolism, and carbon fixation genes. **(B)** Carbon degradation genes. **(C)** Nitrogen metabolism genes. **(D)** Sulfur metabolism genes. The columns and deviation bars indicate the mean ± SD.

Supplementary Figure S4 Temporal distance-decay analysis of functional genes and the bacterial community during shrimp culture.

## Footnotes

^1^ http://ieg.ou.edu/microarray/

## References

[ref1] Akanji M. A. Olagoke O. A. Oloyede O. B. (1993). Effect of chronic consumption of metabisulphite on the integrity of the rat kidney cellular system. Toxicology 81, 173–179. doi: 10.1016/0300-483X(93)90010-P, PMID: 8212023

[ref2] Anderson M. J. (2001). A new method for non-parametric multivariate analysis of variance. Austral Ecol. 26, 32–46. doi: 10.1111/j.1442-9993.2001.01070.pp.x

[ref3] Aufdenkampe A. K. Mayorga E. Raymond P. A. Melack J. M. Doney S. C. Alin S. R.. (2011). Riverine coupling of biogeochemical cycles between land, oceans, and atmosphere. Front. Ecol. Environ. 9, 53–60. doi: 10.1890/100014

[ref4] Baldwin D. S. Mitchell A. (2012). Impact of sulfate pollution on anaerobic biogeochemical cycles in a wetland sediment. Water Res. 46, 965–974. doi: 10.1016/j.watres.2011.11.065, PMID: 22204939

[ref5] Benjamini Y. Hochberg Y. (1995). Controlling the false discovery rate: a practical and powerful approach to multiple testing. J. R. Stat. Soc. Series B Stat. Methodol. 57, 289–300. doi: 10.1111/j.2517-6161.1995.tb02031.x

[ref6] Bonerba E. Ceci E. Bozzo G. Di Pinto A. Tantillo G. (2013). Analysis of the sulphite content in shrimps and prawns. Ital. J. Food Saf. 2:e18. doi: 10.4081/ijfs.2013.959

[ref7] Boroughs L. K. DeBerardinis R. J. (2015). Metabolic pathways promoting cancer cell survival and growth. Nat. Cell Biol. 17, 351–359. doi: 10.1038/ncb3124, PMID: 25774832 PMC4939711

[ref8] Canfield D. E. Farquhar J. (2012). “The global sulfur cycle,” in Fundamentals of Geobiology. eds. Knoll A. H. Canfield D. E. Konhauser K. O. (Hoboken, NJ: Wiley), 49–64.

[ref9] Carrero-Colón M. Nakatsu Cindy H. Konopka A. (2006). Effect of nutrient periodicity on microbial community dynamics. Appl. Environ. Microbiol. 72, 3175–3183. doi: 10.1128/AEM.72.5.3175-3183.2006, PMID: 16672455 PMC1472307

[ref10] Doney S. C. Mahowald N. Lima I. Feely R. A. Mackenzie F. T. Lamarque J.-F.. (2007). Impact of anthropogenic atmospheric nitrogen and sulfur deposition on ocean acidification and the inorganic carbon system. Proc. Natl. Acad. Sci. U. S. A. 104, 14580–14585. doi: 10.1073/pnas.0702218104, PMID: 17804807 PMC1965482

[ref11] Edgar R. C. (2010). Search and clustering orders of magnitude faster than BLAST. Bioinformatics 26, 2460–2461. doi: 10.1093/bioinformatics/btq461, PMID: 20709691

[ref12] Edgar R. C. (2013). UPARSE: highly accurate OTU sequences from microbial amplicon reads. Nat. Methods 10, 996–998. doi: 10.1038/NMETH.2604, PMID: 23955772

[ref13] Edgar R. C. Haas B. J. Clemente J. C. Quince C. Knight R. (2011). UCHIME improves sensitivity and speed of chimera detection. Bioinformatics 27, 2194–2200. doi: 10.1093/bioinformatics/btr381, PMID: 21700674 PMC3150044

[ref14] FAO (2017). Global and China aquaculture production. Available at: https://www.fao.org/fishery/facp/CHN/en (Accessed November 18, 2021).

[ref15] Gao Y. Ding J. Yuan M. Chiariello N. Docherty K. Field C.. (2021). Long-term warming in a Mediterranean-type grassland affects soil bacterial functional potential but not bacterial taxonomic composition. NPJ Biofilms Microbiomes 7:17. doi: 10.1038/s41522-021-00187-7, PMID: 33558544 PMC7870951

[ref16] Gao D. Liu M. Hou L. Derrick Y. F. L. Wang W. Li X.. (2019). Effects of shrimp-aquaculture reclamation on sediment nitrate dissimilatory reduction processes in a coastal wetland of southeastern China. Environ. Pollut. 255:113219. doi: 10.1016/j.envpol.2019.113219, PMID: 31539849

[ref17] Gerner-Smidt P. Keiser-Nielsen H. Dorsch M. Stackebrandt E. Ursing J. Blom J.. (1994). Lautropia mirabilis gen. Nov., sp. nov., a gram-negative motile coccus with unusual morphology isolated from the human mouth. Microbiology 140, 1787–1797. doi: 10.1099/13500872-140-7-1787, PMID: 8075812

[ref18] Gokarn R. R. Eiteman M. A. Martin S. A. Eriksson K. E. (1997). Production of succinate from glucose, cellobiose, and various cellulosic materials by the ruminal anaerobic bacteria Fibrobacter succinogenes and Ruminococcus flavefaciens. Appl. Biochem. Biotechnol. 68, 69–80. doi: 10.1007/BF02785981, PMID: 9373931

[ref19] He Z. Deng Y. Xu M. Li J. Liang J. Xiong J.. (2020). Microbial functional genes commonly respond to elevated carbon dioxide. Environ. Int. 144:106068. doi: 10.1016/j.envint.2020.106068, PMID: 32871382

[ref20] Hou D. Huang Z. Zeng S. Liu J. Wei D. Deng X.. (2017). Environmental factors shape water microbial community structure and function in shrimp cultural enclosure ecosystems. Front. Microbiol. 8:2359. doi: 10.3389/fmicb.2017.02359, PMID: 29238333 PMC5712584

[ref21] Hou D. Huang Z. Zeng S. Liu J. Weng S. He J. (2018). Comparative analysis of the bacterial community compositions of the shrimp intestine, surrounding water and sediment. J. Appl. Microbiol. 125, 792–799. doi: 10.1111/jam.13919, PMID: 29777622

[ref22] Hou D. Zhou R. Zeng S. Wei D. Deng X. Xing C.. (2021). Stochastic processes shape the bacterial community assembly in shrimp cultural pond sediments. Appl. Microbiol. Biotechnol. 105, 5013–5022. doi: 10.1007/s00253-021-11378-9, PMID: 34097120

[ref23] Huang Z. Hou D. Zhou R. Zeng S. Xing C. Wei D.. (2021). Environmental water and sediment microbial communities shape intestine microbiota for host health: the central dogma in an anthropogenic aquaculture ecosystem. Front. Microbiol. 12:772149. doi: 10.3389/fmicb.2021.772149, PMID: 34795658 PMC8593368

[ref24] Huang Z. J. Zeng S. Z. Xiong J. B. Hou D. W. Zhou R. J. Xing C. G.. (2020). Microecological Koch's postulates reveal that intestinal microbiota dysbiosis contributes to shrimp white feces syndrome. Microbiome 8:32. doi: 10.1186/s40168-020-00802-3, PMID: 32156316 PMC7065354

[ref25] Kabil O. Vitvitsky V. Banerjee R. (2014). Sulfur as a signaling nutrient through hydrogen sulfide. Annu. Rev. Nutr. 34, 171–205. doi: 10.1146/annurev-nutr-071813-105654, PMID: 25033061 PMC4684266

[ref26] Kaster K. M. Grigoriyan A. Jennneman G. Voordouw G. (2007). Effect of nitrate and nitrite on sulfide production by two thermophilic, sulfate-reducing enrichments from an oil field in the North Sea. Appl. Microbiol. Biotechnol. 75, 195–203. doi: 10.1007/s00253-006-0796-5, PMID: 17245576

[ref27] Kembel S. W. Cowan P. D. Helmus M. R. Cornwell W. K. Morlon H. Ackerly D. D.. (2010). Picante: R tools for integrating phylogenies and ecology. Bioinformatics 26, 1463–1464. doi: 10.1093/bioinformatics/btq166, PMID: 20395285

[ref28] Kissel D. E. Bock B. R. Ogles C. Z. (2020). Thoughts on acidification of soils by nitrogen and sulfur fertilizers. Agrosystem. Geosci. Environ. 3:e20060. doi: 10.1002/agg2.20060

[ref29] Kleindienst S. Herbst F.-A. Stagars M. von Netzer F. von Bergen M. Seifert J.. (2014). Diverse sulfate-reducing bacteria of the Desulfosarcina/Desulfococcus clade are the key alkane degraders at marine seeps. ISME J. 8, 2029–2044. doi: 10.1038/ismej.2014.51, PMID: 24722631 PMC4184016

[ref30] Kondratyev K. Y. Varotsos C. A. Krapivin V. F. Savinykh V. P. (2004). “Biogeochemical cycles of pollutants in the environment,” in Global Ecodynamics: A Multidimensional Analysis. eds. Kondratyev K. Y. Varotsos C. A. Krapivin V. F. Savinykh V. P. (Berlin Heidelberg: Springer), 381–480.

[ref31] Kuypers M. M. M. Marchant H. K. Kartal B. (2018). The microbial nitrogen-cycling network. Nat. Rev. Microbiol. 16, 263–276. doi: 10.1038/nrmicro.2018.9, PMID: 29398704

[ref32] Langenheder S. Lindström E. S. Tranvik L. J. (2005). Weak coupling between community composition and functioning of aquatic bacteria. Limnol. Oceanogr. 50, 957–967. doi: 10.4319/lo.2005.50.3.0957

[ref33] Louca S. Parfrey L. W. Doebeli M. (2016). Decoupling function and taxonomy in the global ocean microbiome. Science 353, 1272–1277. doi: 10.1126/science.aaf4507, PMID: 27634532

[ref34] Louca S. Polz M. F. Mazel F. Albright M. B. N. Huber J. A. O’Connor M. I.. (2018). Function and functional redundancy in microbial systems. Nat. Ecol. Evol. 2, 936–943. doi: 10.1038/s41559-018-0519-1, PMID: 29662222

[ref35] Ma B. Stirling E. Liu Y. Zhao K. Zhou J. Singh B. K.. (2021). Soil biogeochemical cycle couplings inferred from a function-taxon network. Research 2021:7102769. doi: 10.34133/2021/7102769, PMID: 33796862 PMC7978035

[ref36] Magoc T. Salzberg S. L. (2011). FLASH: fast length adjustment of short reads to improve genome assemblies. Bioinformatics 27, 2957–2963. doi: 10.1093/bioinformatics/btr507, PMID: 21903629 PMC3198573

[ref37] Mello B. L. Alessi A. M. McQueen-Mason S. Bruce N. C. Polikarpov I. (2016). Nutrient availability shapes the microbial community structure in sugarcane bagasse compost-derived consortia. Sci. Rep. 6:38781. doi: 10.1038/srep38781, PMID: 27941835 PMC5150498

[ref38] Morrison E. Newman S. Bae H. S. He Z. Zhou J. Reddy K. R.. (2016). Microbial genetic and enzymatic responses to an anthropogenic phosphorus gradient within a subtropical peatland. Geoderma 268, 119–127. doi: 10.1016/j.geoderma.2016.01.008

[ref39] Naylor R. L. Hardy R. W. Buschmann A. H. Bush S. R. Cao L. Klinger D. H.. (2021). A 20-year retrospective review of global aquaculture. Nature 591, 551–563. doi: 10.1038/s41586-021-03308-6, PMID: 33762770

[ref40] Neis E. P. J. G. Dejong C. H. C. Rensen S. S. (2015). The role of microbial amino acid metabolism in host metabolism. Nutrients 7, 2930–2946. doi: 10.3390/nu7042930, PMID: 25894657 PMC4425181

[ref41] Ning D. Deng Y. Tiedje J. M. Zhou J. (2019). A general framework for quantitatively assessing ecological stochasticity. Proc. Natl. Acad. Sci. U. S. A. 116, 16892–16898. doi: 10.1073/pnas.1904623116, PMID: 31391302 PMC6708315

[ref42] Nriagu J. O. Coker R. D. (1983). Sulphur in sediments chronicles past changes in lake acidification. Nature 303, 692–694. doi: 10.1038/303692a0

[ref43] Ochoa-Hueso R. Delgado-Baquerizo M. Risch A. C. Schrama M. Morriën E. Barmentlo S. H.. (2021). Ecosystem coupling: a unifying framework to understand the functioning and recovery of ecosystems. One Earth 4, 951–966. doi: 10.1016/j.oneear.2021.06.011

[ref44] Rosseel Y. (2012). Lavaan: an R package for structural equation modeling. J. Stat. Softw. 48, 1–36. doi: 10.18637/jss.v048.i02

[ref45] Toju H. Peay K. G. Yamamichi M. Narisawa K. Hiruma K. Naito K.. (2018). Core microbiomes for sustainable agroecosystems. Nat. Plants 4, 247–257. doi: 10.1038/s41477-018-0139-4, PMID: 29725101

[ref46] Tylianakis J. M. Didham R. K. Bascompte J. Wardle D. A. (2008). Global change and species interactions in terrestrial ecosystems. Ecol. Lett. 11, 1351–1363. doi: 10.1111/j.1461-0248.2008.01250.x, PMID: 19062363

[ref47] Valencia-Castañeda G. Frías-Espericueta M. G. Vanegas-Pérez R. C. Pérez-Ramírez J. A. Chávez-Sánchez M. C. Páez-Osuna F. (2018). Acute toxicity of ammonia, nitrite and nitrate to shrimp Litopenaeus vannamei Postlarvae in low-salinity water. Bull. Environ. Contam. Toxicol. 101, 229–234. doi: 10.1007/s00128-018-2355-z, PMID: 29754207

[ref48] Valiente-Banuet A. Aizen M. A. Alcántara J. M. Arroyo J. Cocucci A. Galetti M.. (2015). Beyond species loss: the extinction of ecological interactions in a changing world. Funct. Ecol. 29, 299–307. doi: 10.1111/1365-2435.12356

[ref49] Vreeland R. H. Litchfield C. D. Martin E. L. Elliot E. (1980). Halomonas elongata, a new genus and species of extremely salt-tolerant bacteria. Int. J. Syst. Evol. Microbiol. 30, 485–495. doi: 10.1099/00207713-30-2-485

[ref50] Wardle D. A. Bardgett R. D. Klironomos J. N. Setala H. van der Putten W. H. Wall D. H. (2004). Ecological linkages between aboveground and belowground biota. Science 304, 1629–1633. doi: 10.1126/science.1094875, PMID: 15192218

[ref51] Warren Y. A. Citron D. M. Merriam C. V. Goldstein E. J. C. (2005). Biochemical differentiation and comparison of Desulfovibrio species and other phenotypically similar genera. J. Clin. Microbiol. 43, 4041–4045. doi: 10.1128/JCM.43.8.4041-4045.2005, PMID: 16081948 PMC1233901

[ref52] Wei D. Zeng S. Hou D. Zhou R. Xing C. Deng X.. (2021). Community diversity and abundance of ammonia-oxidizing archaea and bacteria in shrimp pond sediment at different culture stages. J. Appl. Microbiol. 130, 1442–1455. doi: 10.1111/jam.14846, PMID: 33021028

[ref53] Wubs E. R. J. van der Putten W. H. Bosch M. Bezemer T. M. (2016). Soil inoculation steers restoration of terrestrial ecosystems. Nat. Plants 2:16107. doi: 10.1038/nplants.2016.107, PMID: 27398907

[ref54] Xu K. Shi W. Zhang K. Liu S. Xie Z. (2021). Synthesis and nitrite/sulfite electrochemical response investigation of fluorene-based cross-linked polyisocyanide. Macromol. Mater. Eng. 306:2100173. doi: 10.1002/mame.202100173

[ref55] Yergeau E. Bezemer T. M. Hedlund K. Mortimer S. R. Kowalchuk G. A. Van Der Putten W. H. (2010). Influences of space, soil, nematodes and plants on microbial community composition of chalk grassland soils. Environ. Microbiol. 12, 2096–2106. doi: 10.1111/j.1462-2920.2009.02053.x, PMID: 21966905

[ref56] You Y. Wang J. Huang X. Tang Z. Liu S. Sun O. J. (2014). Relating microbial community structure to functioning in forest soil organic carbon transformation and turnover. Ecol. Evol. 4, 633–647. doi: 10.1002/ece3.969, PMID: 25035803 PMC4098142

[ref57] Yuan Z. Y. Chen H. Y. H. (2015). Decoupling of nitrogen and phosphorus in terrestrial plants associated with global changes. Nat. Clim. Chang. 5, 465–469. doi: 10.1038/nclimate2549

[ref58] Zhou R. Wang H. Wei D. Zeng S. Hou D. Weng S.. (2021). Bacterial and eukaryotic community interactions might contribute to shrimp culture pond soil ecosystem at different culture stages. Soil Ecol. Lett. doi: 10.1007/s42832-021-0082-6

[ref59] Zhou J. Xing J. (2021). Haloalkaliphilic denitrifiers-dependent sulfate-reducing bacteria thrive in nitrate-enriched environments. Water Res. 201:117354. doi: 10.1016/j.watres.2021.117354, PMID: 34157573

[ref60] Zhou R. Zeng S. Hou D. Liu J. Weng S. He J.. (2020). Temporal variation of antibiotic resistance genes carried by culturable bacteria in the shrimp hepatopancreas and shrimp culture pond water. Ecotoxicol. Environ. Saf. 199:110738. doi: 10.1016/j.ecoenv.2020.110738, PMID: 32447139

[ref61] Zhu Y.-G. Gillings M. Penuelas J. (2020). Integrating biomedical, ecological, and sustainability sciences to manage emerging infectious diseases. One Earth 3, 23–26. doi: 10.1016/j.oneear.2020.06.004, PMID: 34173527 PMC7340083

[ref62] Zhu Y. G. Zhao Y. Li B. Huang C. L. Zhang S. Y. Yu S.. (2017). Continental-scale pollution of estuaries with antibiotic resistance genes. Nat. Microbiol. 2:16270. doi: 10.1038/nmicrobiol.2016.270, PMID: 28134918

[ref63] Zhu Y. G. Zhao Y. Zhu D. Gillings M. Penuelas J. Ok Y. S.. (2019). Soil biota, antimicrobial resistance and planetary health. Environ. Int. 131:105059. doi: 10.1016/j.envint.2019.105059, PMID: 31374443

