# Supplementary figures and images for: Sedimentary Nitrogen and Sulfur Reduction Functional-Couplings Interplay With the Microbial Community of Anthropogenic Shrimp Culture Pond Ecosystem
Source: Front Microbiol. 2022 Mar 4;13:830777. doi: 10.3389/fmicb.2022.830777 (PMC8931606; doi:10.3389/fmicb.2022.830777)

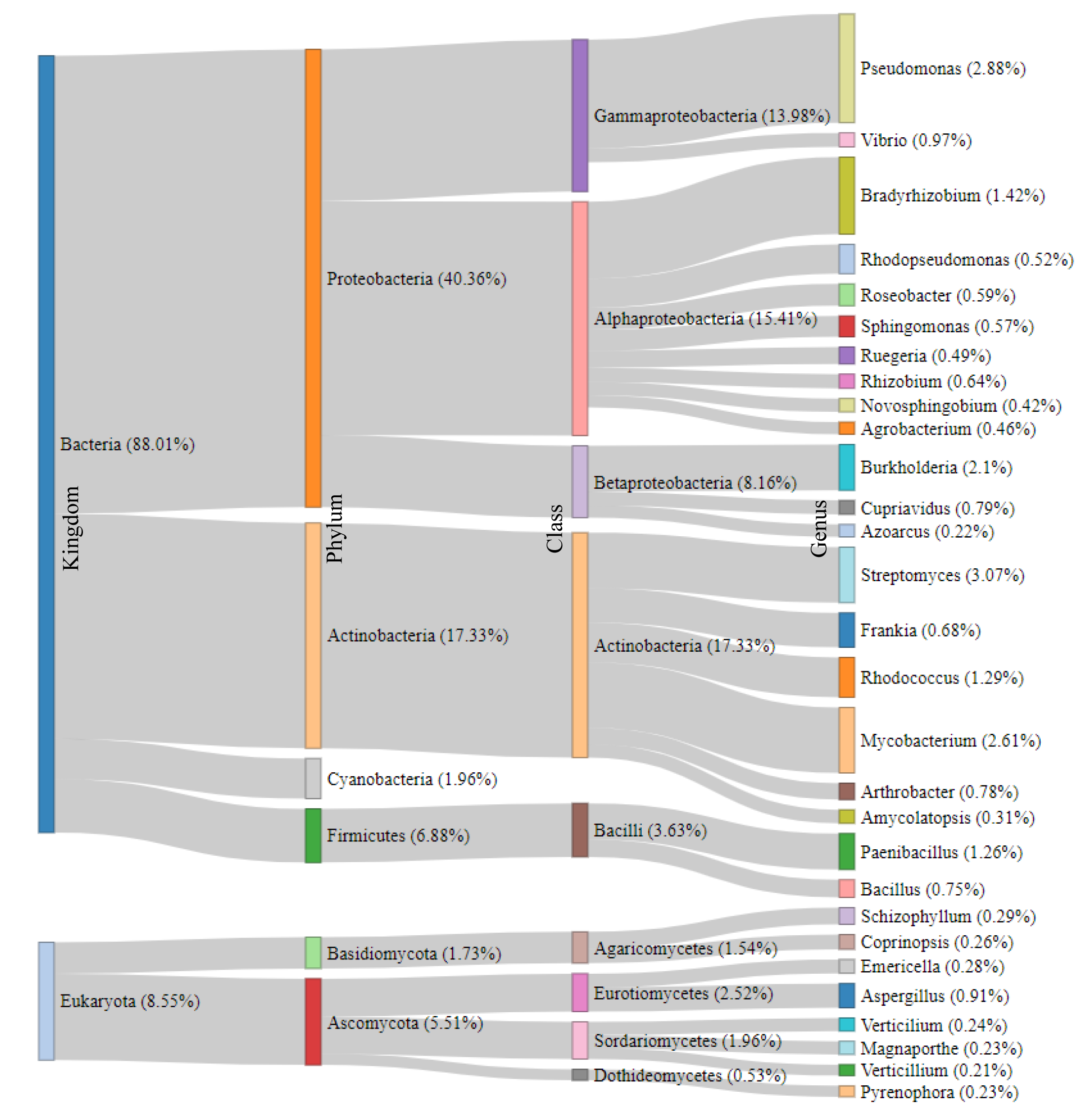

Supplement: Supplementary Figure S1 — Taxonomic composition of functional gene host. [file Image_1.TIFF]

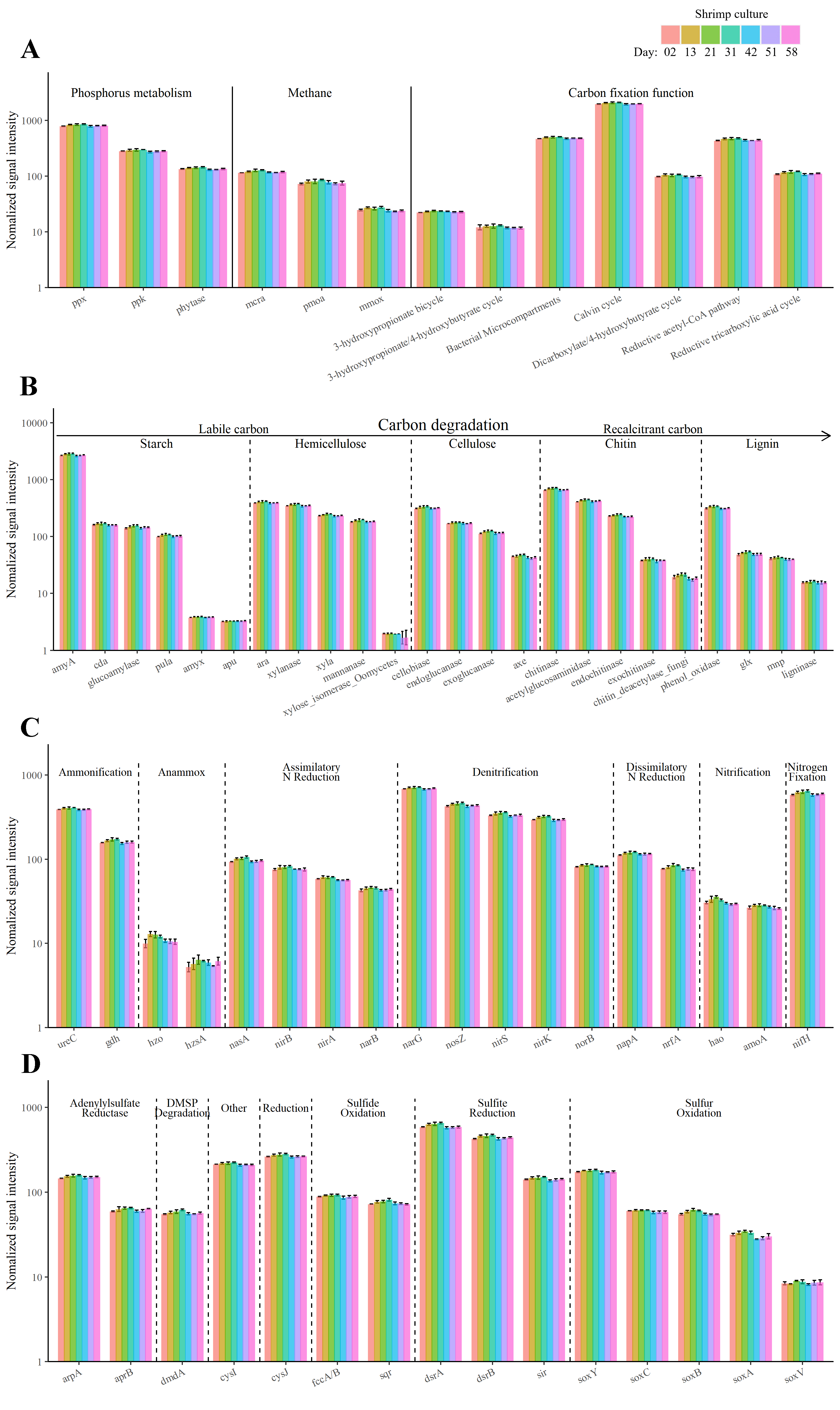

Supplement: Supplementary Figure S3 — The signal intensity of biogeochemical functional genes in GeoChip, (A) phosphorous, methane metabolism, and carbon fixation genes. (B) Carbon degradation genes. (C) Nitrogen metabolism genes. (D) Sulfur metabolism genes. The columns and deviation bars indicate the mean ± SD. [file Image_3.TIF]

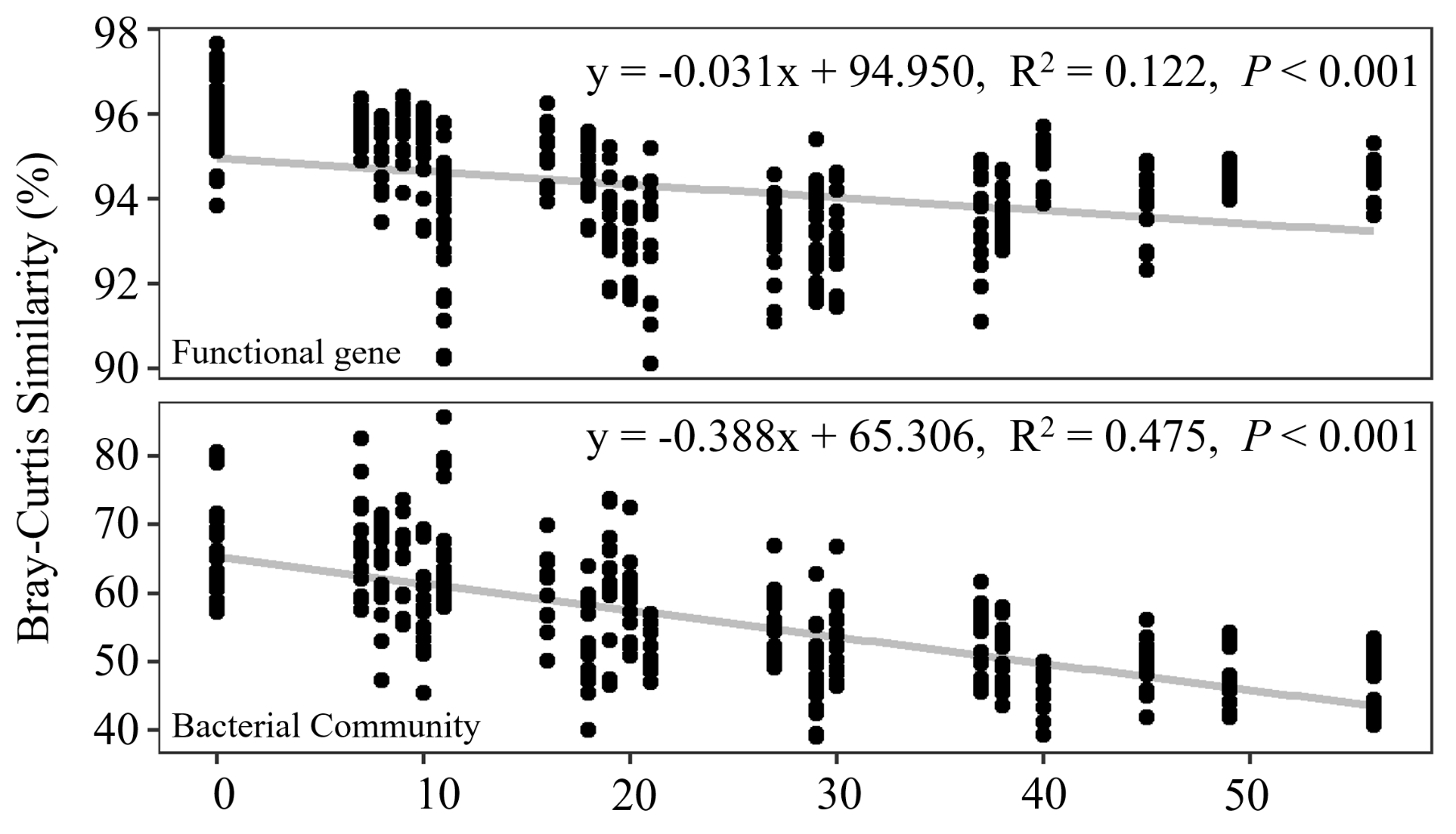

Supplement: Supplementary Figure S4 — Temporal distance-decay analysis of functional genes and the bacterial community during shrimp culture. [file Image_4.TIFF]
